# Race and Ethnicity in Cardiovascular Disease Risk Prediction for Multiethnic Populations: Insights From Global Guidelines

**DOI:** 10.1016/j.jacasi.2025.10.027

**Published:** 2026-07-01

**Authors:** Ann Hui Ching, Hazirah Mohamad, Satveer Kaur-Gill, Reuben Ng, Nathan D. Wong, Eugene Yang, Howard Bauchner, Mayank Dalakoti

**Affiliations:** a University of Oxford School of Anthropology and Museum Ethnography, Oxford, United Kingdom; b Dalla Lana School of Public Health, University of Toronto, Toronto, Ontario, Canada; c Department of Communication Studies, University of Nebraska–Lincoln, Lincoln, Nebraska, USA; d Lee Kuan Yew School of Public Policy, National University of Singapore, Singapore, Singapore; e Heart Disease Prevention Program, Mary and Steve Wen Cardiovascular Division, University of California, Irvine, School of Medicine, Irvine, California, USA; f Division of Cardiology, University of Washington School of Medicine, Seattle, Washington, USA; g Chobanian & Avedisian School of Medicine, Boston University, Boston, Massachusetts, USA; h British Heart Foundation Cardiovascular Epidemiology Unit, Department of Public Health and Primary Care, University of Cambridge, Cambridge, United Kingdom; i Cardiovascular Metabolic Translational Research Program, National University of Singapore, Singapore, Singapore; j Department of Cardiology, National University Heart Centre Singapore, Singapore, Singapore

**Keywords:** clinical practice guidelines, preventive cardiology, race/ethnicity, risk prediction models, social determinants of health

## Abstract

Cardiovascular disease is the leading cause of death worldwide, with certain racial/ethnic groups facing higher risks. Global clinical guidelines for the prevention of cardiovascular disease vary in their approach to addressing racial/ethnic differences among patients. The authors compare the American Heart Association’s 2024 PREVENT (Predicting Risk of Cardiovascular Disease Events) equations, the European Society of Cardiology’s 2021 Systematic Coronary Risk Evaluation 2 model, and the Singapore-modified Framingham risk score, with a focus on their differing approaches to race and ethnicity. The PREVENT model removes race and ethnicity as a factor, instead incorporating the Social Deprivation Index to address social determinants of health. SCORE2 introduces multiplier factors for different ethnicities, while the Singapore-modified Framingham risk score retains ethnicity as a variable. Race-neutral models such as PREVENT aim to avoid reinforcing race as a biological construct while still accounting for social determinants of health that are highly correlated with race. In Asia, the path toward race-neutral risk prediction begins with strengthening data infrastructure and increasing participation in clinical trials to ensure adequate representation.

## Highlights

• Ethnic disparities in CVD persist despite effective prevention tools.
• We compare how the CVD guidelines of the AHA, ESC, and Singapore use race/ethnicity.
• Race-based models imply biology; race-neutral ones may miss the impact of racism.
• Future models can improve risk prediction via ancestry, SDoH, and access factors.

Cardiovascular disease (CVD) is the leading cause of mortality, accounting for almost 21 million deaths in 2021.^1^ Despite the availability of effective, low-cost strategies for the primary prevention of CVD and for treating hypertension and hypercholesterolemia, cardiovascular risk factors (CVRFs) remain under-recognized and undertreated.^2^ Certain ethnic groups, such as South Asian individuals, bear a disproportionately high burden of atherosclerotic CVD (ASCVD), with higher rates of diagnosis as well as increased morbidity and mortality.^3^ Recently, the American Heart Association (AHA) and the European Society of Cardiology (ESC) released updated guidelines for risk stratification in the primary prevention and screening of CVD. These updates incorporate distinct approaches aimed at managing ethnic disparities in cardiovascular health. In the 2024 PREVENT (Predicting Risk of Cardiovascular Disease Events) equations, the AHA has removed race/ethnicity as a variable, replacing it with place of residence (ZIP code) as a proxy for social determinants of health (SDoH). SDoH encompass factors such as economic stability, access to education, health care quality, neighborhood and built environment, and social community context.^4^ These factors are closely linked to the morbidity and mortality associated with CVD.^5^ The ESC addresses SDoH in its 2021 SCORE2 (Systematic Coronary Risk Evaluation 2), using national registries and cohort studies to classify European nations into region-specific risk prediction groups. The American College of Cardiology (ACC)/AHA guideline on the primary prevention of CVD^6^ and the ESC guidelines on CVD prevention in clinical practice^2^ are the 2 most widely cited guidelines for CVD screening, with almost 7,000 and 3,000 citations, respectively. In Singapore, the latest guideline for coronary heart disease (CHD) is the Singapore-modified Framingham risk score 2023 (SG-FRS-2023), which includes ethnicity as a key variable. Within Asia, Singapore is a useful model to evaluate determinants of chronic disease development in Asia, because individuals representing the major Asian ethnic groups of Chinese, Indian, and Malay live in a small country with good access to health care.^7^

Asia is a diverse region characterized by wide variations in culture, socioeconomic status, and health literacy. With increasing intranational and international migration,^8^ professional organizations must adapt clinical practice guidelines to reflect the growing diversity within countries. Although these recommendations are often intended primarily for audiences in the United States and Europe, many other countries modify internationally recognized, evidence-based standards into their respective guidelines. Although discussions of race/ethnicity in medicine are often U.S.-centric, many countries have diverse populations.^9^ Recently, SCORE2 has been adapted to Asia, though only at a national level,^10^ and it does not address the granularity of ethnic heterogeneity within a country. For instance, data from the United Kingdom show that even within individuals grouped together as South Asian,^3^ higher ASCVD risk has been observed among those of Bangladeshi origin, followed by Pakistani and Indian adults.^11^ In the United States, immigrants from China, Japan, and Korea are homogenized as East Asian, as individual ethnicities are not adequately represented among major U.S. prospective CVD cohort studies that form the basis of the ACC/AHA ASCVD pooled cohort equations (PCE) and other risk calculators.^12^ In this review, we specifically explore how race/ethnicity can be integrated into CVD risk assessment through the varied approaches of the 2 most cited CVD prevention guidelines in comparison with the SG-FRS-2023, an adaptation of the 1998 U.S.-based Framingham risk score.^13^ A key challenge is the tension between using race/ethnicity as a variable, potentially reinforcing harmful misconceptions about race as biologically defined, and the risk of disregarding it entirely, which may obscure the impact of systemic racism.^14^ Rather than a prescriptive solution, our goal is to illuminate key issues as public health, cardiovascular, and other professionals design clinical practice guidelines for their own nations, tailored to the complexities of their national contexts. This discussion is important to the adaptation of screening, prevention, and management strategies into local practices to effectively enable preventive cardiology efforts in Asia.^15^

Race and ethnicity are distinct concepts. Race is a social-political construct.^16^ In the context of North America and Europe, race emerged from a colonial history, used to justify hierarchies, such as settler over native and master over enslaved, by categorizing people according to physical characteristics.^17^ Race has historically been linked to external traits such as skin color and facial features. With the emergence of genetics in the 20th century, race was mistakenly thought to correspond to genetically distinct and homogeneous populations. Although this idea has since been debunked,^18^ race continues to be used as a proxy for genetic ancestry, with broad genetic clusters aligning with continental regions. In contrast, ethnicity refers to group identity on the basis of shared cultural characteristics.^19^ In this paper, we adopt the terminology as defined in each guideline. Specifically, we use “race/ethnicity” in reference to the PREVENT guidelines and “ethnicity” when discussing the ESC and Singapore guidelines.

## Overview of International Clinical Practice Guidelines on Primary ASCVD Prevention

### PCE

The PCE, introduced in 2013 by the ACC and AHA and updated in the 2019 ACC/AHA guideline on the primary prevention of CVD,^6^ remain the currently endorsed tool in the United States for estimating 10-year risk for ASCVD in adults. The PCE are based on data from National Heart, Lung, and Blood Institute cohort studies that incorporated data from 24,626 individuals (20,338 non-Hispanic White and 4,288 African-American participants).

The PCE stratify risk by sex and race,^20^ specifically, non-Hispanic White, non-Hispanic Black, and “other.” As individuals in the “other” category rely on coefficients derived from non-Hispanic White participants, the PCE overestimate risk for certain populations, including Asian and Hispanic individuals.^20^

### PREVENT

The 2024 PREVENT tool was developed as a race-neutral alternative to the PCE. Although not yet formally incorporated into current ACC/AHA cholesterol management or primary prevention guidelines, PREVENT is intended to inform future updates to cardiovascular risk assessment practices. For example, the recent 2025 hypertension guideline includes PREVENT for identifying who should be considered for antihypertension treatment.^21^ PREVENT estimates 10-year and 30-year sex-stratified risk for total CVD, ASCVD, and heart failure (HF) in U.S. adults.^22^ PREVENT was derived from more than 3 million individuals across 28 different data sets^22^ incorporating both traditional cohort data and large-scale electronic health records, including electronic health records from the Optum Labs Data Warehouse, which alone contributed 94% of the individuals in the derivation data set.^22^

Unlike the PCE, PREVENT includes longer term outcomes of 30-year sex-stratified risk and integrates additional cardiovascular, kidney, and metabolic health variables. In PREVENT, estimated glomerular filtration rate is a mandatory variable in the risk calculation, along with age, total cholesterol, high-density lipoprotein, systolic blood pressure, diabetes diagnosis, current smoking status, body mass index, and the use of lipid-lowering medications (Table 1). Meanwhile, urinary albumin/creatinine ratio, glycated hemoglobin, and the Social Deprivation Index (SDI) are optional variables but are useful for further refinement of risk estimation.^22^

**Table 1:** Summary of Primary CVD Risk Calculators in the United States, Europe, and Singapore

	United States	Europe	Singapore
Most recent scoring system	AHA PREVENT (2023)	ESC SCORE2 (2021) and SCORE2-OP	Singapore MOH SG-FRS-2023
Primary outcome	10-y risk estimates for 30-79 y of age and 30-y risk estimates for 30-59 y of age of fatal and nonfatal ASCVD^a^	10-y risk for CVD events^b^	10-y risk for coronary heart disease
Risk factors	Compulsory • Age • Antihypertensive medication • BMI • Diabetes diagnosis • eGFR • HDL • Lipid-lowering medication • SBP • Smoking status • Total cholesterol Optional • HbA_1c_ • uACR • SDI using 5-digit zip code	Compulsory • Age • Country of residence • Non-HDL cholesterol • SBP • Smoking status	Compulsory • Age • HDL • SBP • Smoking status • Total cholesterol
Use of race and/or ethnicity in risk calculator	Race and ethnicity	Ethnicity	Ethnicity
Definition of an individual’s ethnic identity	Definitions range from self-reported or as communities that are predominantly White/Black	Not defined	Ethnicity as listed on national registration identity card according to the CMIO model^c^
Accounting for interethnic disparities in ASCVD	Inclusion of the SDI to estimate social determinants of health and consideration of CKM health	Accounts for interethnic disparities using multiplying factors^d^ derived from QRISK3, an ASCVD prediction algorithm developed in the United Kingdom	Individual’s score is assessed based on sex stratified tables with ethnicity-specific columns^e^

AHA = American Heart Association; ASCVD = atherosclerotic cardiovascular disease; BMI = body mass index; CKM = cardiovascular, kidney, and metabolic; CMIO = Chinese-Malay-Indian-other; CVD = cardiovascular disease; eGFR = estimated glomerular filtration rate; ESC = European Society of Cardiology; HbA1c = glycated hemoglobin; HDL = high-density lipoprotein; HF = heart failure; MOH = Ministry of Health; PREVENT = Predicting Risk of Cardiovascular Disease Events; SBP = systolic blood pressure; SCORE2 = Systematic Coronary Risk Evaluation 2; SDI = Social Deprivation Index; SG-FRS-2023 = Singapore-modified Framingham risk score 2023; uACR = urinary albumin/creatinine ratio.

a Separate risk calculators for HF, ASCVD, and total CVD (combined HF and ASCVD).

b Events include ASCVD, hypertensive disease, arrhythmias, HF, abdominal aortic aneurysms, sudden death within 24 hours of symptom onset, and nonfatal myocardial infarction.

c Individual’s ethnicity follows the ethnicity of either parent. For individuals born to interethnic couples, there is an option for double-barreled race.

d Suggested multiplying factors are South Asian (1.3 for Indians and Bangladeshis, 1.7 for Pakistanis), other Asian (1.1), Black Caribbean (0.85), and Black African and Chinese (0.7).

e Individuals who are “other race” are interpreted using the Chinese specific tables.

Although the PCE incorporate race as a factor stratifying risk, PREVENT is race neutral and does not include race in its risk stratification. The AHA decided not to include race in the development of PREVENT,^23^ citing concerns that incorporating race could imply inherent racial/ethnic differences and reinforce race as a biological construct.^23^ Instead, the PREVENT uses 5-digit zip code (for U.S. residents) as a measure of the SDI, a surrogate for SDoH. The SDI captures neighborhood-level indicators such as poverty rates, educational attainment, single-parent household, and rental housing, which reflect some of broader social conditions influencing health outcomes. Race/ethnicity structures our social and individual lived experiences and is associated with adverse SDoH, representing a key driver of adverse CVD outcomes.^23^ Hence, the use of the SDI could address the root cause of interethnic disparities in CVD. However, using zip codes as a proxy for SDoH may not capture other important factors, such as community connections and access to quality health care. Although the SDI in PREVENT was well calibrated for non-Hispanic White and non-Hispanic Black individuals, similar calibration for other racial/ethnic groups, including non-Hispanic Asian and Hispanic populations, was not performed.

### SCORE2

The ESC 2021 CVD prevention guideline^2^ uses the SCORE2 model to estimate sex-based risk for developing CVD in healthy adults. SCORE2 builds upon the original SCORE model, which was first introduced in 2016. The SCORE model was developed using pooled data from cohort studies across 12 European countries, primarily to address the overestimation of CVD risk observed in certain European populations when the Framingham risk score was applied.^24^ Unlike SCORE, which predicts only the risk for fatal cardiovascular events in individuals without histories of CVD,^24^ SCORE2 expands the definition of CVD to include nonfatal myocardial infarction, stroke, and HF. SCORE2 also includes the creation of a separate risk estimation chart for individuals 70 years and older. Moreover, SCORE2 calibrates sex-specific models for the varying risk levels across different European countries by categorizing European nations into 4 distinct risk groups (low, moderate, high, and very high) on the basis of World Health Organization CVD mortality rates, ensuring more accurate, region-specific risk assessment.^25^

The SCORE2 Asia-Pacific algorithm is a recalibrated version of the original SCORE2 cardiovascular risk prediction model, tailored specifically to the age-, sex-, and region-specific risk factor distributions and CVD incidence in Asia-Pacific populations.^10^ Similar to the original SCORE2, countries throughout the Asia-Pacific region are divided into low, moderate, high, and very high risk. Although SCORE2 was developed using data from Western cohorts, SCORE2 Asia-Pacific retains its original predictive coefficients but adjusts baseline risk estimates using local data on CVD mortality, risk factor prevalence, and disease incidence patterns, sourced from regional data sets and World Health Organization estimates. The recalibration process involved regional adaptation using aggregate data, external validation with independent cohorts, and comparison with locally derived models.

The 2021 ESC guidelines recognize significant heterogeneity in the prevalence of CVD and associated risk factors among immigrants from India, China, North Africa, and Pakistan.^2^ However, ethnicity data were not available in the cohort studies and registries used to develop and recalibrate the SCORE2 models. Instead, the guidelines suggest using ethnicity-specific relative risks in CVD risk calculations. Citing the example of QRISK3, a 10-year CVD risk prediction algorithm developed in the United Kingdom,^26^ the ESC proposes refining risk scores by incorporating ethnicity-specific multiplying factors (Table 1). However, these approaches may not fully capture the heterogeneity of risk within a given ethnic group. Additionally, given the vast diversity of global populations, no risk score can comprehensively represent all ethnic groups.

## Singapore: A Multiethnic Population

Singapore is a highly developed, multiethnic nation of nearly 6 million people in Southeast Asia.^27^ Despite allocating only about 6% of its annual gross domestic product to health care, Singapore consistently achieves excellent health outcomes. In 2023, ASCVD accounted for 30.9% of all deaths in Singapore.^28^ Singapore’s population is officially classified under the Chinese-Malay-Indian-other model, which groups residents into 4 main ethnicities for administrative and policy purposes.^29^ An individual’s ethnicity is determined at birth on the basis of the ethnicity of either the father or the mother. Those of mixed-race heritage can now be registered with a double-barreled racial designation, representing both ethnicities.^30^ In 2020, Chinese people made up the largest ethnic group, constituting 74% of the population, followed by Malays at 14% and Indians at 9%.^31^ The “other” category, referring to individuals of who are not of Chinese, Malay, or Indian descent, accounts for 3% of the population.^32^ The Chinese-Malay-Indian-other model, which traces back to the British colonial era,^33^ continues to shape public health data collection and population-based risk assessments, including the development of the SG-FRS-2023.

### Ministry of Health clinical practice guidelines and SG-FRS-2023

The SG-FRS-2023 model^34^ is a sex- and ethnicity-stratified risk model used to estimate the 10-year risk for CHD in healthy individuals. This model updates the 2016 Singapore Adult Treatment Panel III, which was introduced in the 2011 Ministry of Health guidelines for the management of lipid disorders.^35^ The Singapore Adult Treatment Panel III is derived from the Framingham risk score for CHD,^13^ where points are assigned on the basis of factors such as age, smoking status, total cholesterol, high-density lipoprotein cholesterol, and systolic blood pressure. In the Framingham model, calculated risk scores are interpreted using sex-based tables. To tailor this model to the Singaporean population, ethnicity was added as an additional variable for interpreting risk scores. The SG-FRS-2023 uses more recent data from a population-based cohort study conducted in Singapore between 2004 and 2010^7^ to recalibrate the Singapore Adult Treatment Panel III model, which originally used data from 1982 and 1995.^36^ Compared with the Singapore Adult Treatment Panel III, the SG-FRS-2023 demonstrated improved accuracy, as the earlier model overestimated the 10-year ASCVD risk, with predicted events nearly twice as high as the actual events observed across both sexes and all ethnicities.^34^ However, as the SG-FS-2023 model does not include prediction of stroke, HF, or CVD death due to non-CHD causes, notably HF, these are important considerations when interpreting prediction from this model compared with the PREVENT and ESC calculators.

### Ethnicity and CVD in Singapore

Ethnicity plays a key role in shaping the perception of CVD risk and public health campaigns in Singapore. According to the Singapore Myocardial Infarction Registry report in 2020, both Indian and Malay populations experienced higher incidences of acute myocardial infarction (AMI) and associated mortality compared with the Chinese population.^33^ From 2007 to 2014, whereas the incidence of ASCVD remained stable among Chinese individuals, it increased among both Indian and Malay groups.^31^ Singapore is geographically small, with a resource-rich health care system with no significant ethnic differences in key metrics that affect AMI morbidity and mortality, such as rates of percutaneous coronary intervention and symptom-to-balloon time.^31^ Some studies suggest that the higher incidence and mortality rates of AMI in these ethnic minorities may be linked to a greater burden of CVRFs. For example, the prevalence of type 2 diabetes among adults older than 60 years is 60% among Indian people, 50% among Malay people, and 25% among Chinese people.^37^ The National Population Health Survey 2020 also highlighted a higher prevalence of hypertension among Malay and Chinese individuals compared with Indian residents and a higher prevalence of overweight and obesity among Malay and Indian individuals compared with Chinese individuals.^38^

Adverse SDoH are associated with higher morbidity and mortality from CVD but also do not fully explain ethnic disparities. In Singapore, the Malay population faces lower levels of educational attainment,^32^ a higher likelihood of living in public housing,^39^ and lower monthly household incomes compared with Chinese and Indian ethnic groups.^39^ Such factors, importantly, are not captured when Asians are aggregated together or considered as an “other” group when used in CVD risk prediction scores. Yet even after adjusting for factors such as income, education, employment, and housing type, Malay ethnicity is still associated with higher risk for mortality from out-of-hospital AMI^40^ compared with Chinese and Indian groups. This may reflect the inability to adjust for all factors that contribute to CVD. In contrast, although Indian individuals shares similar socioeconomic status with Chinese individuals,^39^ Indian ethnicity remains an independent risk factor for AMI, particularly among younger individuals.^41^ The limited availability of accurate information on family history of premature CVD to be incorporated into these risk scores is also a limitation that may significantly affect risk in certain individuals.

The disparities in CVRFs and the prevalence and severity of ASCVD among different ethnicities in Singapore has prompted research into alternative explanations,^42^ including serum biomarkers^43^ and physiological differences potentially linked to unique genetic factors.^44^ Culture, defined as the set of norms, beliefs, and behaviors inherent to a particular ethnic group,^45^ has also been suggested as a contributing factor to the higher susceptibility of Malay and Indian individuals in Singapore to ASCVD. For example, public health messaging has sometimes attributed the increased rates of ASCVD among Malays in Singapore as being linked to the consumption of traditional foods high in coconut milk, sugar, and saturated fats.^46^ However, such a simplistic view risks oversimplifying culture and at worst reinforces negative stereotypes and unfairly places the blame for poor health on ethnic minorities themselves.^47^

## Discussion

A standardized approach to incorporating race and ethnicity for CVD risk assessment in clinical practice guidelines is lacking on a global scale. With increasing migration both within and between nations, it is essential to develop a clearer understanding of how to appropriately account for racial and ethnic diversity at both the individual and population levels (Central Illustration). Unlike the PREVENT and SCORE2, SG-FRS-2023 only measures the risk for CHD, rather than total CVD. The AHA has removed race/ethnicity as a specific variable in CVD risk stratification, opting instead to include factors that reflect the higher burden of comorbidities, such as chronic kidney disease, severity of diabetes (eg, glycated hemoglobin), and SDoH through the SDI among ethnic minorities. In contrast, the SG-FRS-2023 continues to use ethnicity as a category in interpreting risk.

**Central Illustration:**
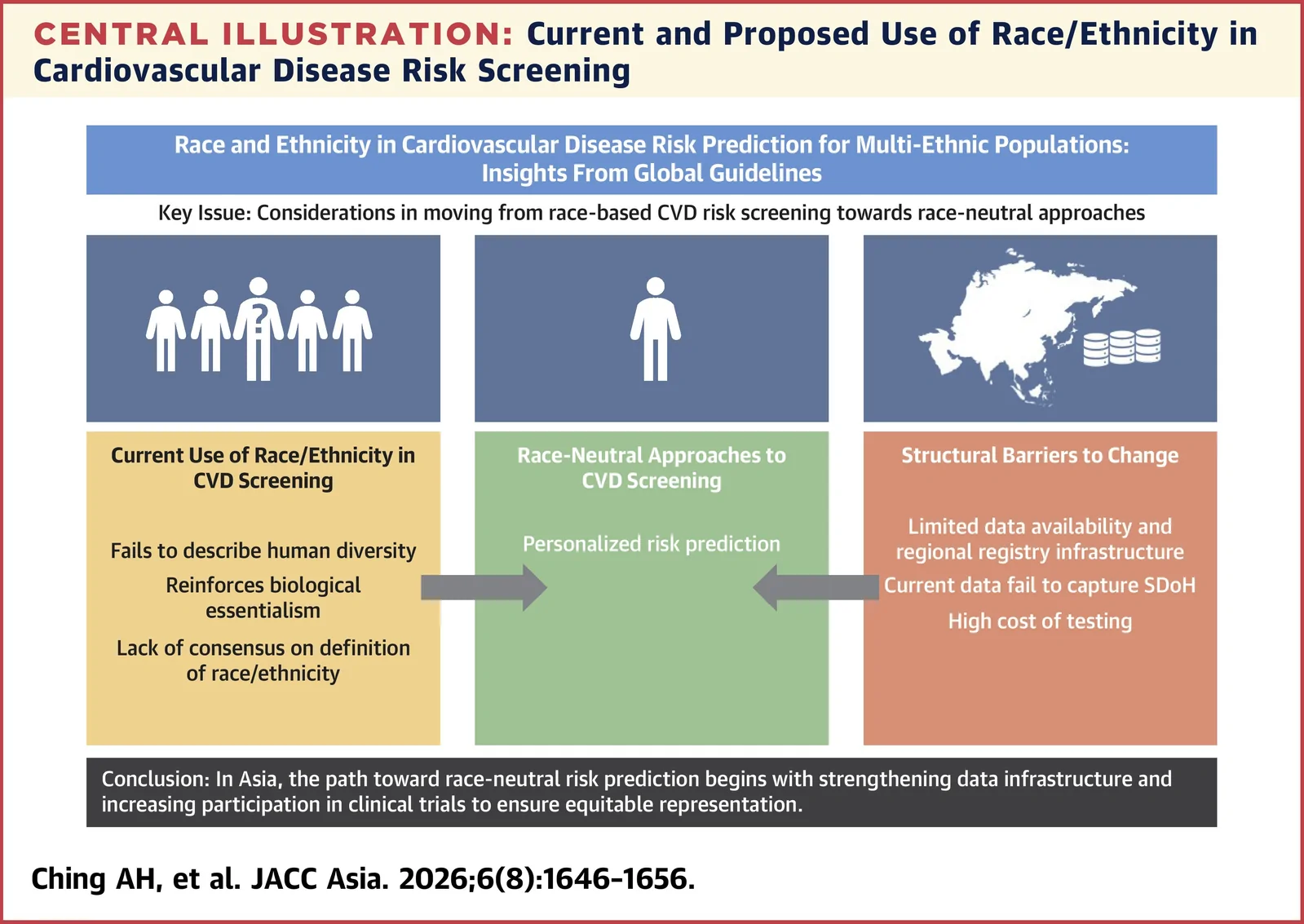
Current and Proposed Use of Race/Ethnicity in Cardiovascular Disease Risk Screening This illustration summarizes the transition from race/ethnicity-based cardiovascular disease risk screening to race-neutral models. The central panel depicts race-neutral approaches emphasizing individualized risk assessment through data-driven models. The left and right panels highlight issues with current race-based methods and the systemic obstacles in the conceptual shift from race-based to race-neutral frameworks. CVD = cardiovascular disease; SDoH = social determinants of health.

The decision to include or exclude race/ethnicity in clinical guidelines has significant implications for how diseases are classified and treated. The same clinical profile can yield markedly different risk estimates depending on the cardiovascular risk score used. For example, in a hypothetical case of a 45-year-old patient presenting for their first primary CVD screening, PREVENT and SCORE2 Asia-Pacific estimated lower risks for both CHD and total CVD compared with SG-FRS-2023 across all 3 ethnicities, especially for patients with high-risk factors (Table 2). Although the PREVENT model has removed race/ethnicity, risk calculation models still exhibit racial and ethnic calibration differences.^22^ For example, the model tends to underestimate ASCVD risk for non-Hispanic Black individuals^22^ and overestimate HF risk for non-Hispanic Asian individuals.^22^ As a result, this could reduce eligibility for statin and antihypertensive therapies for an estimated 15.8 million adults in the United States, with African Americans being most affected.^48^ Understanding the consequences of omitting race/ethnicity in prediction models is essential for addressing population health and may inadvertently exacerbate existing racial disparities.

**Table 2:** Variability in Primary CVD Risk Estimates Across Calculators for a 45-Year-Old Nonobese Singaporean

	Male	Female	Diabetic (Newly Diagnosed, Male)
Low risk Nonsmoker SBP 120 mm Hg Total cholesterol 150 mg/dL HDL 60 mg/dL	PREVENT • Total CVD 1.37% • CHD only 0.34% SCORE2 Asia-Pacific • Total CVD 5% SG-FRS-2023 (CHD) ◦ Chinese: <1% ◦ Malay: <1% ◦ Indian: <1%	PREVENT • Total CVD 0.95% • CHD only 0.22% SCORE2 Asia-Pacific • Total CVD 2% SG-FRS-2023 (CHD) ◦ Chinese: <1% ◦ Malay: <1% ◦ Indian: <1%	PREVENT • Total CVD 3.63% • CHD only 0.89% Risk calculated by SCORE2 Asia-Pacific and SG-FRS-2023 same as in column for male
High risk Current smoker SBP 160 mm Hg Total cholesterol 200 mg/dL HDL 40 mg/dL	PREVENT • Total CVD 3.63% • CHD only 0.89% SCORE2 Asia-Pacific • Total CVD 14% SG-FRS-2023 (CHD) • Chinese: 9% • Malay: 14% • Indian: 19%	PREVENT • Total CVD 6.96% • CHD only 2.36% SCORE2 Asia-Pacific • Total CVD 7% SG-FRS-2023 (CHD) • Chinese: 4% • Malay: 7% • Indian: 10%	PREVENT • Total CVD 18.92% • CHD only 7.17% Risk calculated by SCORE2 Asia-Pacific and SG-FRS-2023 same as in column for male

CHD = coronary heart disease; other abbreviations as in Table 1.

The limitations of existing racial categories in capturing diversity may necessitate a race-neutral approach as stated in the Central Illustration. First, disease epidemiology can vary significantly among individuals who are categorized as the same race/ethnicity across different continents. For example, Black people have a lower risk for CVD compared with non-Hispanic White people in the United Kingdom,^26^ whereas the opposite is observed in the United States.^49^ Second, as the population of mixed-race individuals continues to grow,^50^ understanding their health outcomes becomes increasingly important. For instance, among Asian and Pacific Islander populations in California, mixed-race groups showed higher rates of CVD compared with single-race Native Hawaiians and other Pacific Islanders.^51^ Third, smaller ethnic minority groups are sometimes combined with larger groups in a single category. In the SG-FRS-2023, individuals identified as “others” are classified under the Chinese category. Similarly in the United States, South Asians are categorized as Asian Americans, yet their significantly higher risk for ASCVD is often underestimated by existing risk scoring systems.^3^

Existing tools to account for associations between SDoH or genetic ancestry and race/ethnicity in relation to increased CVD risk remain insufficient for creating truly race-neutral guidance. Although race is a social construct, it can reflect the lived and embodied effects of racism,^21^^,^^52^ which have measurable biological consequences.^53^ Advances in epigenetics suggest that disease patterns and physical differences are influenced not only by genetic ancestry but also by the historical experiences of racism.^54^ To address the adverse SDoH associated with ethnic minority status, PREVENT included the SDI, an important first step. However, the inclusion of the SDI to the required variables in PREVENT led to an overestimation of CVD and HF risk among Hispanic, Asian, and other ethnic groups.^22^ Future iterations may benefit from emerging tools that incorporate more specific SDoH factors, such as geospatial information^55^ or variables to assess social connections.^56^ However, incorporating additional variables could reduce its practicality and ease of use, which is likely to be a challenge with PREVENT.^57^

Personalized medicine holds the potential to incorporate biomolecular testing for CVD risk assessment, potentially eliminating the need for ethnicity-based models. In the PREVENT model, integrating cardiovascular, kidney, and metabolic health variables better captures the disproportionate CVD burden among ethnic minorities. However, the inclusion of optional biomarkers that are widely used in clinical practice, such as glycated hemoglobin, urinary albumin/creatinine ratio, and the SDI, worsened the calibration of PREVENT for ASCVD in Asian and other groups and for HF in Hispanic, Asian, and other groups compared with the models using core risk factors alone.^22^

Beyond these routinely measured parameters, numerous emerging biomarkers show promise for refining CVD risk prediction. Current risk assessment tools are designed largely to predict the occurrence of clinical CVD events, but expanding detection to include individuals with subclinical atherosclerosis would enable earlier, more targeted preventive strategies and would significantly improve risk prediction for the purposes of allocating preventive therapy, including among Asian patients.^58^ Lipoprotein(a),^59^ a low-density lipoprotein–like particle, that is more than 90% genetically determined is known to drive ASCVD through plaque accumulation and has been recommended in universal screening by the European Atherosclerosis Society,^60^ the Canadian Cardiovascular Society,^61^ the Lipid Association of India,^53^ and the National Lipid Association.^62^ In the move toward screening used for both primary and secondary ASCVD, high-sensitivity cardiac troponin T and N-terminal pro–B-type natriuretic peptide have shown high prognostic value in identifying individuals without prior ASCVD at similar risk as those with prior ASCVD. High-sensitivity cardiac troponin T and N-terminal pro–B-type natriuretic peptide consistently enhance risk discrimination in diverse populations and have been consistently shown to improve CVD risk prediction in a number of study populations with diverse backgrounds, in one instance achieving performance comparable with adding all 7 conventional predictors in established models. Imaging can also be considered in cardiovascular risk assessment. Given the earlier onset and more aggressive nature of disease among Indians, the Lipid Association of India recommends significantly more aggressive screening.^53^ The Lipid Association of India emphasizes shifting from 10-year ASCVD risk estimation to assessing lifetime risk and advises the broader use of subclinical atherosclerosis assessment (including coronary artery calcium scoring, carotid and femoral ultrasound, and ankle-brachial index) beginning at 30 years of age. However, access to such testing can vary greatly and is limited among underserved patients. For instance, despite formal recommendation by the European Atherosclerosis Society, limited awareness, variable costs and coverage, and a lack of currently available pharmacotherapies have limited the widespread implementation of lipoprotein(a).^63^ Moreover, there are concerns that advanced biomolecular testing could inadvertently worsen racial and ethnic disparities, as ethnic majorities often have better access to genomic testing.^63^^,^^64^

National-level organizations face substantial practical challenges in collecting biomolecular data and establishing the advanced data infrastructure required to analyze risk factors linked to race, including SDoH and genetic ancestry. Although race is a social construct, it reflects lived experiences of racism that can have measurable biological consequences. In contexts in which such data infrastructure remains limited, retaining ethnic classifications remains valuable, not as biological proxies but as benchmarks for identifying disparities and as markers of sociocultural and structural contexts shaping health outcomes. Within Asia, only 4 countries (China, India, Malaysia, and Singapore) maintain registries on CHD or HF,^65^ and 3 centers in Pakistan and 2 centers in Vietnam participate in the ACC National Cardiovascular Data Registry.^66^ Even though 60% of global cardiometabolic disease exists in the Asia-Pacific region, only 8.3% of participants in clinical trials published in *JAMA*, *The Lancet*, and the *New England Journal of Medicine* over the past decade were identified as “Asian.”^67^ Broad ethnic categories such as South Asian, Southeast Asian, and East Asian may obscure within-country diversity^68^ and require subgroup analyses that reflect national contexts and capture residual heterogeneity in the development of national-level risk calculators.^69^^,^^70^ Addressing these gaps requires a multipronged strategy to strengthen clinical trial design, conduct, and leadership in the Asia-Pacific region through investments in research training, regional funding, transparent reporting, and equitable representation.^71^

## Conclusions

Predicting CVD risk in multiethnic populations presents significant challenges because of the inconsistent use of terms such as “race/ethnicity” in international guidelines, the limitations of existing data, and the complex biological implications of experienced racism. The inclusion of race in guidelines often fails to capture the spectrum of diversity and may inadvertently reinforce the flawed notion of race as a biologically homogenous category. However, race-neutral guidelines, although crucial, risk overlooking the nuanced biological effects of racism and complex factors associated with race, such as genetic ancestry and SDoH. The PREVENT model represents a significant step forward in race-neutral risk stratification, but further research is essential to understand its impact across diverse groups, including how the performance of risk scores can be improved in general. In Asia, the path toward race-neutral risk prediction begins with strengthening data infrastructure and increasing participation in clinical trials to ensure equitable representation.

## Funding Support and Author Disclosures

This research is supported by the Singapore Ministry of Health’s National Medical Research Council under its National Medical Research Council Open Fund – Large Collaborative Grant (OFLCG22may-0010), Project RESET: Redirecting Immune, Lipid and Metabolic Drivers of Early Cardiovascular Disease. Dr Yang is supported by the University of Washington Medicine Asian Health Initiative and the Carl and Renée Behnke Endowed Chair for Asian Health. Dr Yang is an advisory board member for Idorsia, Mineralys, Qure.ai, and Sky Labs; is a consultant for Genentech; has received honoraria from the ACC; and has received research grants from Microsoft Research. All other authors have reported that they have no relationships relevant to the contents of this paper to disclose.
